# Supplementary material for: The Comprehensive Characterization of B7-H3 Expression in the Tumor Microenvironment of Lung Squamous Cell Carcinoma: A Retrospective Study
Source: Cancers (Basel). 2024 Jun 4;16(11):2140. doi: 10.3390/cancers16112140 (PMC11171371; doi:10.3390/cancers16112140)
Supplement: Supplementary file 1 [file cancers-16-02140-s001.zip › Table S1_uni_multivariate.pdf]

Supplementary Table S1 Univariate and multivariate analyses of clinicopathological factors related to overall survival.

**Univariate analysis**				**Multivariate analysis**		
		n	P value	Hazard ratio	95% CI	P value
B7-H3(tumor)	High	46	0.034	0.3855	0.1519,0.9786	0.04492
	Low	57				
B7-H3(stroma)	High	48	0.693			
	Low	55				
Gender	Male	77	0.705			
	Female	26				
Age	>70	51	0.299			
	<=70	52				
Stage	II	67	0.449			
	III	36				
Brinkman Index	>600	76	0.858			
	<=600	27				
ly	+	25	0.04	0.9954	0.9954,5.1900	0.05129
	-	78				
v	+	81	0.284			
	-	22				
pl	+	60	0.572			
	-	43				
pm	+	8	0.062			
	-	95				
