# Supplementary figures and images for: The Comprehensive Characterization of B7-H3 Expression in the Tumor Microenvironment of Lung Squamous Cell Carcinoma: A Retrospective Study
Source: Cancers (Basel). 2024 Jun 4;16(11):2140. doi: 10.3390/cancers16112140 (PMC11171371; doi:10.3390/cancers16112140)

# Supplementary Figure S1 Asakawa et al.

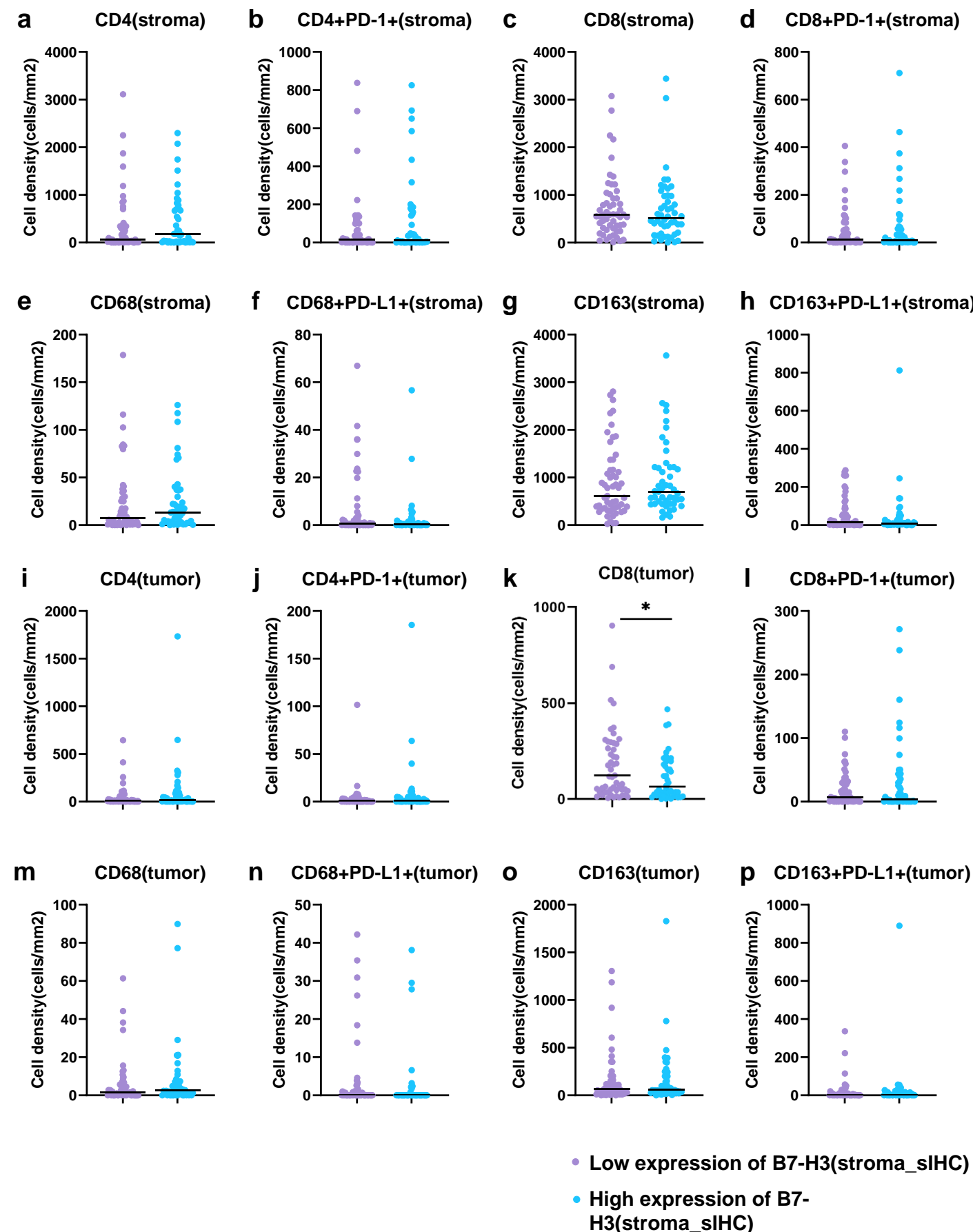

Supplement: Supplementary file 1 [file cancers-16-02140-s001.zip › Figure S1_B7_stroma_comparison_230512.pdf]
